# Two or More Synchronous Combination of Noninvasive Tests to Increase Accuracy of Liver Fibrosis Assessement in Chronic Hepatitis C; Results From a Cohort of 446 Patients

**DOI:** 10.5812/hepatmon.853

**Published:** 2012-03-28

**Authors:** Dana Crisan, Corina Radu, Monica Lupsor, Zeno Sparchez, Mircea Dan Grigorescu, Mircea Grigorescu

**Affiliations:** 1 Third Medical Clinic, Regional Institute of Gastroenterology and Hepatology, “Iuliu Hatieganu” University of Medicine and Pharmacy, Cluj-Napoca, Romania

**Keywords:** Hepatitis C, Chronic, Aminopyrine, Liver Cirrhosis

## Abstract

**Background:**

The prediction of fibrosis is an essential part of the assessment and management of patients with chronic liver disease. Non-invasive tests (NITs) have a number of advantages over the traditional standard of fibrosis assessment by liver biopsy, including safety, cost-effectiveness, and widespread accessibility.

**Objectives:**

The aim of this study was to determine the accuracy of certain biomarkers and transient elastography (TE) alone or in combination to predict the stage of liver fibrosis in chronic hepatitis C (CHC). Also, we examined whether the combination of certain biomarkers and TE could increase the diagnostic accuracy of liver fibrosis assessment.

**Patients and Method:**

A total of 446 patients who were previously diagnosed with CHC were included in the study. In the study group, 6 blood-based scores (APRI, Forns, Fib-4, Hepascore, FibroTest, and Fibrometer) were calculated, and TE was performed to validate the stage of fibrosis, compared with liver biopsy (LB) as the standard.

**Results:**

Significant fibrosis (F ≥ 2) was predicted with an AUROC of 0.727, 0.680, 0.714, 0.778, 0.688, 0.797, and 0.751 for the APRI, Forns, Fib-4, FibroTest, Hepascore, and Fibrometer scores and TE (Fibroscan), respectively. Severe fibrosis (F ≥ 3) was predicted, with AUROCs ranging between 0.705 and 0.811 for Hepascore and Fibrometer, respectively. Of the biomarkers, Fibrometer had the highest AUROC value in predicting both significant and severe fibrosis. The combination of APRI or FIB-4 with Fibrometer increased the diagnostic accuracy for significant fibrosis (from 69.07 to 82.27 for APRI, P = 0.001 and from 57.74 to 81.33, P = 0.001 for Fib-4). Combining APRI or Fib-4 with TE also increased the diagnostic accuracy (from 69.07 to 80.70%, P = 0.001 for APRI and from 57.74 to 81.33%, P = 0.001 for Fib-4) for significant fibrosis. The association that included Fibrotest was also reliable for the improvement of diagnostic accuracy. These combinations were more accurate or the assessment of severe fibrosis.

**Conclusions:**

The synchronous association between a simple, inexpensive score and a complex but expensive score or TE increases the diagnostic accuracy of non-invasive methods for the assessment of liver fibrosis stage.

## 1. Background

Progressive hepatic ﬁbrosis is a characteristic of chronic liver diseases, and its significance derives from the evolution toward cirrhosis with subsequent complications. Therefore, the assessment of fibrosis in chronic liver diseases not only is a determinant of the prognosis but also establishes the optimal timing of therapy, screening, and surveillance strategies and may also predict the treatment response. Historically, the stage of fibrosis has been assessed by liver biopsy, and despite its inconveniences, this method remains the standard of evaluation. Liver biopsy (LB) also has the advantages of suggesting the etiology and assessing potential disease cofactors, such as hepatic steatosis, hepatocellular iron, and necroinﬂammatory activity. On the other hand, liver biopsy is an invasive method that is painful and has a risk of bleeding [1]. Furthermore, liver biopsy is costly and brings into discussion the sampling error [2] and differences in pathologist interpretation [3]. These limitations have led to the search for alternative, non-invasive tests for ﬁbrosis assessment, including clinical and serum biomarker algorithms. The number of serum biomarker algorithms for liver ﬁbrosis has increased signiﬁcantly over the past decade, and they are beginning to be incorporated into routine clinical practice. Biomarkers of ﬁbrosis are typically divided into indirect and direct markers of ﬁbrogenesis and ﬁbrolysis. Indirect markers include simple, routine tests, such as aminotransferases, platelet count, and albumin or prothrombin time [4]. Direct markers include serum levels of matrix metalloproteinases and hyaluronic acid and cytokines, such as tumor necrosis factor-a (TNF-α) and transforming growth factor-β (TGF-β), which are more directly involved in the molecular pathogenesis of ﬁbrogenesis and ﬁbrolysis [5].

In addition to the advantages of noninvasive biomarkers, there are some pitfalls that render these algorithms imperfect. Many of the models that are used for the assessment of liver fibrosis have good accuracy in determining advanced fibrosis and cirrhosis, but the main challenge remains the determination of mid-levels of ﬁbrosis. The same problem also exists for TE. The components of scores or algorithms can also contribute to the imperfection of these diagnostic methods. In general, models that include direct markers of ﬁbrogenesis (Fibrotest, Hepascore, Fibrospect) tend to have higher accuracy in predicting signiﬁcant ﬁbrosis than those that are based on indirect measures (APRI, Forns, FIB-4), although comparative studies have often failed to detect statistically signiﬁcant differences. Transient elastography is a novel, rapid, noninvasive, and reproducible method for measuring liver stiffness. Preliminary reports suggest that FS accurately predicts hepatic fibrosis in patients with chronic hepatitis C.

## 2. Objectives

The aim of this study was to evaluate the diagnostic performance and reliability of non-invasive serological and imagistic methods for the assessment of significant and severe fibrosis in chronic hepatitis C (CHC) compared to liver biopsy. Also, we evaluated the possibility of an increase in diagnostic accuracy when we combined different scores or when we added an imagistic method to the biomarkers to assess the severity of liver fibrosis.

## 3. Patients and Method

This study prospectively included 446 consecutive patients who were previously diagnosed with CHC and underwent liver biopsy at the 3rd Medical Clinic, Cluj-Napoca, Romania. Chronic hepatitis C infection was defined as the presence of anti-HCV for at least 6 months and positivity for HCV RNA. We excluded patients with other etiologies of chronic liver disease, such as hepatitis B, autoimmune liver disease, Wilson disease, hemochromatosis, α1-antitripsin deficiency, and HIV infection; and patients with a history of hepatotoxic or steatosis-inducing drug use or alcohol consumption of more than 20 g/day for women and 30 g/day for men. All patients were naïve for antiviral treatment. The study was performed in full accordance with the Declaration of Human Rights (Helsinki, 1975) and its revisions. Complete, comprehensive, and clear informed consent was obtained from the patients. The study was approved by the local ethical committee of the Clinical Emergency Hospital "Prof. Dr. Octavian Fodor" in Cluj-Napoca. All patients signed prior informed consent forms on inclusion into the study.

### 3.1. Laboratory Investigations

All patients underwent hematological, biochemical, and virological examinations on the day of their participation. In order to exclude biliary obstruction and the presence of liver focal lesions, abdominal ultrasonography was performed for all patients on inclusion into study. A blood sample was obtained after 8 hours of overnight fasting for routine investigations (aminotransferases, AST, ALT, fasting plasma glucose, platelet count, gamma glutamyl transpeptidase, bilirubin, cholesterol, urea, prothrombin time). All assessments were made on an automatic analyzer (Konelab 30 I - Thermo Electron Corp Finland). All patients were infected with HCV genotype 1. Serum HCV RNA was measured by PCR (Cobas Amplicor HCV 2.0 version, Roche). Serum samples from each patient were stored at -70°C for further biochemical analysis. All biochemical tests and their scores were assessed without knowledge of the liver biopsy results. The serum biochemical markers α2 macroglobulin (α2-MG), haptoglobin, and apolipoprotein A1 were assessed by nephelometry. The following non-invasive markers were determined in the study group, per published formulas: AST-to-platelet ratio index (APRI) [6] Forns' index [7], and Fib-4 [8]. The formulas for the serological scores were:

- APRI = [{AST (IU/l)/ ALT_ULN (IU/l)} × 100]/ platelet count (10(9)/L)

- FiB-4 = [age (y) × AST (IU/L)]/ [platelet count (10(9)/L) × ALT (IU/L) 1/2]

- Forns' Index = 7.811 - 3.131 × ln [platelet count (10(9)/L)] + 0.781 × ln[GGT(IU/L)] + 3.467 × ln [age (y)] - 0.014 [cholesterol (mg/dL)]

The following blood tests were also evaluated: Fibrotest® [9], Fibrometer [10], Hepascore® [11], calculated according to the patented formulas or computed on the dedicated websites.

### 3.2. Liver Stiffness Measurement

Transient elastography (TE) (Fibroscan ©, Echosens, Paris, France) [12] was performed on the right liver lobe by a well-trained and experienced operator. TE was considered valid if 10 measurements were obtained with a success rate of > 60% and an interquartile range < 30% of the median. Patients with invalid scores were excluded from the analysis.

### 3.3. Morphopathological Study

Liver biopsies, obtained under ultrasonographic guidance and stained with hematoxylin-eosin and Masson's trichrome, were assessed blindly per the METAVIR scoring system [13] by an expert pathologist. According to the METAVIR system, fibrosis was staged on a scale from F0 to F4, as follows: F0: no fibrosis; F1: portal fibrosis, without septa; F2: few septa; F3: many septa without cirrhosis; and F4: cirrhosis. F0 and F1 were considered insignificant fibrosis, whereas scores of F2-F4 were considered significant fibrosis; F3-F4 was considered severe fibrosis.

### 3.4. Statistical Analysis

Comparisons between groups were made using student's t-test for continuous variables with normal distribution and χ(2) test for categorical variables. Values were expressed as mean ± SD. Continuous variables with non-normal distribution were expressed as median and 25-75th percentiles, and the differences were analyzed by Mann-Whitney test. Correlations between scores and fibrosis stage variables were established using Spearman's rank correlation. Variables that achieved statistical significance in the univariate analysis were included in an ordinal regression analysis to evaluate the independent factors that were associated with significant fibrosis. P < 0.05 was considered statistically significant. Binary analysis was performed using receiver operator characteristic (ROC) curves, separating patients into 2 groups based on fibrosis stage (F0-1 vs. F2-4 and F0-2 vs. F3-4). Cutoff values for each test in the cohort were established, where sensitivity and specificity were maximal. The diagnostic value of each test was assessed through the area under the ROC (AUROC), and it was also expressed as a percentage for the diagnostic accuracy. Standardization of AUROC was performed based on the prevalence of fibrosis stages (DANA method) [14]. The sensibility (Se), specificity (Sp), positive predictive value (PPV), negative predictive value (NPV), positive likelihood ratio (PLR) and negative likelihood ratio (NLR) of the serum biomarkers in predicting different stages of fibrosis were calculated using ROC analysis. De Long's method was used for AUROC comparison. The diagnostic value of the combinations of different scores was assessed using tests for dichotomic variables. The agreement between methods was assessed using McNemar test for the categorial variables and the diagnostic accuracy based on true positive, true negative, false positive and false negative data. We used MedCalc® 9.3.9.0. and SPSS version 15.0 (SPSS Inc. Chicago, IL, USA) for the statistical analysis.

## 4. Results

The clinical, biochemical, and histological data are summarized in (Table 1). The mean age of the study group was 49 year, with a predominance of females (61.59%). The median length of the liver biopsy samples was 11.45 mm, with a mean number of portal spaces of 13.58. No sample had fewer than 6 portal spaces. The fibrosis stage distribution was as follows: F0, n = 30 (6.72%); F1, n = 133 (28.82%); F2, n = 161 (36.09%); F3, n = 81 (18.16%); and F4, n = 41 (9.19%). The output of each marker was compared with the fibrosis stage, divided into 2 groups, depending on significant and severe fibrosis. We compared the differences in scores between the patients with significant fibrosis and mild fibrosis and between those with severe fibrosis and mild-moderate fibrosis, from which we obtained the corresponding AUROCs (Table 2). Liver stiffness ranged from 3 to 27 kPa (median 7.1 [5.450 - 10.125]). The most discriminant cutoff values were determined from the distribution of stiffness values according to fibrosis stage and ROC curves: 7.9 for F ≥ 2 and 9 for F ≥ 3. In patients with stiffness values greater than 7.9 kPa, the likelihood of significant fibrosis was 90.3% (positive predictive value). The AUROC for each test for the diagnosis of significant fibrosis ranged between 0.680 for Forns score and 0.797 for Fibrometer (Table 1). For the discrimination of severe fibrosis, AUROCs of non-invasive tests ranged between 0.737 for Forns score and 0.811 for Fibrometer (Table 2). The comparison of ROC curves demonstrated slight differences in diagnostic performance between indirect and direct biomarker tests and also for the TE.

**Table 1:** Demographic, Laboratory, and Histological Features of Patients With Chronic Hepatitis C

	**Patients (n = 446)**
Gender, No. (%), (median)	170 (38.1)
Males	276 (61.59)
Females	49 (40 - 56)
Age, y, No. (%)	49 (40 - 56)
BMI, kg/m^2^, (median)	26.56 (23.66 - 29.74)
Prothrombin time, s, (median)	99 (89.00 - 108.30)
Total cholesterol, mg/dL, (median)	192.00 (167.00 - 220.50)
Bilirubin, mg/dL, (median)	0.70 (0.54 - 0.89)
AST, IU/L, (median)	50.00 (34.00 - 73.50)
ALT, IU/L, (median)	76.00 (51.00 - 117.50)
GGT, IU/L, (median)	52.00 (34.00 - 88.00)
Urea, mg/dL, (median)	33.00 (27.00 - 41.00)
Platelet count, Giga/L, (median)	210.00 (169.00 - 247.25)
Length of biopsy, mm, mean ± SD, (median)	11.45 ± 0.68
Portal tracts, No., mean ± SD, (median)	13.58 ± 4.74
Liver fibrosis according to METAVIR , No. (%)	
F0	30 (6.72)
F1	133 (28.82)
F2	161 (36.09)
F3	81 (18.16)
F4	41 (9.19)

**Table 2:** The Performance of Non-Invasive Serological Tests and TE Expressed by AUROC in Discriminating Significant and Sever Fibrosis (F0-1 vs. F2-4)

**Score**	**AUC a**	**95% CI**	**Cut-off**	**Se a**	**Sp a**	**PPV a**	**NPV a**	**PLR a**	**NLR a**	**Accuracy**
**Discriminating Significant Fibrosis**										
APRI a	0.727	0.666 to 0.783	0.44	71.92	66.67	77.2	60.2	2.16	0.42	69.03
Forns	0.680	0.616 to 0.739	4.47	79.86	49.45	41.4	60.8	1.58	0.41	67.23
Fib-4	0.714	0.652 to 0.771	1.26	64.38	75.27	80.3	57.4	2.60	0.47	57.74
Hepascore	0.688	0.618 to 0.751	0.34	57.25	72.13	82.3	42.7	2.05	0.59	61.3
Fibro meter	0.797	0.722 to 0.860	0.59	68.75	80.85	88.0	55.9	3.59	0.39	72.72
Fibro Test	0.778	0.722 to 0.828	0.34	64.91	79.52	86.7	52.4	3.17	0.44	73.33
TE	0.751	0.681 to 0.813	7.90	53.72	87.50	90.3	46.7	4.30	0.50	64.55
**Discriminating Sever Fibrosis**										
APRI	0.741	0.681 to 0.796	1.69	61.40	77.47	46.1	86.5	2.73	0.50	72.38
Forns	0.737	0.676 to 0.792	7.3	85.71	48.60	34.3	91.6	1.67	0.29	57.02
FIB-4	0.767	0.708 to 0.819	3.74	63.16	80.77	50.7	87.5	3.28	0.46	76.15
Hepascore	0.705	0.636 to 0.767	0.61	60.66	73.19	50.0	80.8	2.26	0.54	68.84
Fibro meter	0.811	0.737 to 0.871	0.76	80.5	72.5	54.1	90.2	2.93	0.27	71.32
Fibro Test	0.782	0.726 to 0.831	0.54	82.89	63.48	49.2	89.7	2.27	0.27	70.07
TE	0.809	0.743 to 0.864	9.00	69.57	84.73	71.5	88.8	4.56	0.36	79.6

^a^ Abbreviations: APRI, aspartate aminotransferase to platelet ratio index; AUC, area under curve; NLR, negative likelihood ratio; NPV, negative predictive value; PLR, positive likelihood ratio; PPV, positive predictive value; Se, sensitivity; Sp, specificity; TE, transient elastography

In order to improve the diagnostic performance, a combination of 2 or 3 methods was tested. Because APRI and Fib-4 were the simple non-invasive tests that appeared to have better diagnostic accuracy for significant and severe fibrosis, they were chosen to form combinations with complex patented formulas or TE. As Fibrometer and Fibotest had the best accuracy with regard to direct complex scores, they formed paired combinations with APRI and Fib-4. The diagnostic accuracy for significant fibrosis increased significantly when Fibrometer was added to APRI (from 69.03 to 82.27, P = 0.001) and Fib-4 (from 57.74 to 81.33, P = 0.001) (Table 3). Another useful combination for the improvement of significant fibosis was the association of APRI with Fibrotest (diagnostic accuracy increased from 69.03 to 80.70) and Fib-4 with Fibrotest (accuracy increased from 57.74 to 78.44). The association of TE to the simple scores also improved diagnostic accuracy for the prediction of F2. The best diagnostic value was obtained with the combination of APRI and Fibrometer for the discrimination of significant fibrosis.

**Table 3:** Comparison of Diagnostic Accuracy for the Combination of Non-Invasive Tests in Discriminating Significant and Severe Fibrosis (F0-1 vs. F2-4)

**Score**	**Se a**	**Sp a**	**PPV a**	**NPV a**	**Accuracy**	**P value**
**Discriminating Significant Fibrosis**						
APRI a	71.92	66.67	77.2	60.2	69.03	
APRI + Fibrometer	79.24	88.46	93.33	67.64	82.27	0.001
APRI + FibroTest	79.24	88.46	91.30	64.44	80.70	0.001
APRI+TE a	72.91	100	100	56.66	79.68	0.001
Fib-4	64.38	75.27	80.3	57.4	57.74	
Fib-4 + Fibrometer	75.51	92.30	94.87	66.66	81.33	0.001
Fib-4 + FibroTest	75.64	84.21	90.76	62.74	78.44	0.001
Fib-4 + TE	47.61	100	100	56	68.57	0.02
APRI + Fib-4	71.42	73.29	82.56	59.09	72.08	0.36(/APRI)
APRI + Fib-4+Fibrometer	80.00	95.00	97.29	67.85	84.61	0.001/NS b
APRI + Fib-4+FibroTest	73.97	86.60	93.65	65.00	82.52	0.001 /NS c
APRI+Fib-4 + TE	73.80	100	100	57.69	80.70	0.001/NS d
**Discriminating Severe Fibrosis**						
APRI	61.40	77.47	46.1	86.5	72.38	
APRI+Fibrometer	78.26	84.37	64.28	91.52	82.75	0.006
APRI+FibroTest	89.65	77.77	61.90	94.91	81.18	0.02
APRI+TE	73.3	81.71	61.11	92.59	85.71	0.001
Fib-4	63.16	80.77	50.7	87.5	76.15	
Fib-4+Fibrometer	84.21	90.47	72.72	95.00	89.02	0.001
Fib-4+FibroTest	89.28	81.57	64.10	95.38	83.65	0.01
Fib-4+TE	75.00	91.37	70.58	92.98	87.83	0.001
APRI+Fib-4	64.58	81.87	51.66	88.51	75.88	
APRI+Fib-4+Fibrometer	84.21	91.07	76.19	94.44	89.33	0.001/NS b
APRI+Fib-4+FibroTest	88.46	83.18	67.64	94.91	84.94	0.003/NS c
APRI+Fib-4+TE	62.5	100	100	58.62	85.51	0.001/NS d

^a^ Abbreviations: APRI, aspartate aminotransferase to platelet ratio index; PPV, positive predictive value; NPV, negative predictive value; Se, sensitivity; Sp, specificity; TE, transient elastography

^b^ P APRI+ Fib-4/P APRI + Fibro meter

^c^ P APRI+ Fib-4/ P APRI + Fibro Test

^d^ P APRI+ Fib-4/P APRI + TE

Severe fibrosis was also evaluated through simple scores combined with Fibrometer; we noted a significant increase in the diagnostic accuracy for APRI associated with Fibrometer (from 72.38 to 82.75, P = 0.006) and for Fib-4 with Fibrometer (from 76.15 to 89.02, P = 0.001). The algorithm including Fibrotest resulted in nearly the same benefit for the diagnostic of severe fibrosis, not only in combination with APRI (accuracy increased from 72.38 to 81.18) but also added to Fib-4 (accuracy increased from 76.15 to 83.65). The combination with TE was also effective in increasing the diagnostic accuracy of severe fibrosis, reaching statistical significance (APRI+TE, accuracy increased from 72.38 to 85.71, and Fib-4+TE from 76.15 to 87.83) (Table 3). The best diagnostic value was obtained with the combination of Fib-4 and Fibrometer for the discrimination of severe fibrosis. The combination of the two indirect scores, APRI and Fib-4, did not significantly increase the diagnostic accuracy for significant or severe fibrosis (P = 0.55, respectively 0.21). We also evaluated the combination of three markers (two simple scores with Fibrometer, Fibrotest, or TE), but the diagnostic accuracy did not increase compared to former associations (APRI plus Fibrometer or APRI plus TE; Fib-4 plus Fibrometer or Fib-4 plus TE). Nevertheless, the association of three scores, including one of the complex scores (Fibrometer, Fibrotest or TE), and two simple scores had a significantly higher diagnostic accuracy compared to the combination of the two simple indirect tests (Table 3). APRI and Fibrometer agreed on the discrimination of significant fibrosis in 70% of patients. The concordant cases of APRI and Fibrometer were confirmed by LB in 82%. Among 18% of discordant cases, 17% of cases were classified as F < 2 by APRI and Fibrometer and F ≥ 2 by LB, and 1% of cases were classified as F ≥ 2 by APRI and Fibrometer and F < 2 by LB. When Fib-4 and Fibrometer agreed on the discrimination of significant fibrosis, which was the case in 78% of the patients, the concordance with the LB examination was 81%. Of the 19% of discordant cases, 15% was classified as < F2 by blood test and F ≥ 2 by LB and 4% was classified as F ≥ 2 by blood test and F < 2 by LB. When TE was included in the algorithm, we noted an agreement on significant fibrosis of 74.5% between APRI and TE, with a concordance of 86% with LB. In the 14% in whom they were not concordant, 12% of cases were classified as F < 2 by Fib-4 and Fibrometer and F ≥ 3 by LB, and 2% of the cases were classified as F ≥ 2 by blood test and F < 3 by LB. The analysis for the discrimination of severe fibrosis, incorporating APRI or Fib-4 with Fibrometer, revealed similar results with the same tests applied for the discrimination of significant fibrosis.

## 5. Discussion

Biomarkers and TE have been recently developed as alternatives to liver biopsy for the staging of liver fibrosis [15]. Retrospective studies [16][17][18] have compared several of these markers to liver biopsy. The real indicator of liver disease status is a histological analysis of the entire liver, but it is impossible to obtain this in practice; hence, liver biopsy remains "the best but not the gold standard" [19]. Many non-invasive methods have been proposed for the assessment of liver fibrosis. There are mainly two ways to approach the assessment of liver fibrosis: using a single test or a combination of different NITs in so-called "decision-making algorithms": SAFE (Sequential Algorithm for Fibrosis Evaluation) or BA (Bordeaux Algorithm) [20]. As the use of serum biomarkers and TE as individual methods is not the gold standard, in order to increase the diagnostic performance of non-invasive markers of liver fibrosis, researchers have tried to combine them in algorithms. Stepwise combination of APRI and Fibro Test improved the diagnostic performance in CHC, with a consequent decrease for the need of liver biopsy by 50% to 70% [21]. Another study tried to validate SAFE, which detects significant fibrosis and cirrhosis by combining the AST-to-platelet ratio index and Fibrotest-Fibrosure, and concluded that SAFE is a rational and validated method for staging liver fibrosis in hepatitis C [22]. Castera et al. compared the diagnostic accuracy of 2 algorithms (SAFE and a combination between FT and TE based on agreement between these tests) and concluded that both algorithms are effective for non-invasive staging of liver fibrosis in CHC [23].

Our study evaluated the diagnostic performance of APRI, Forns, Fib-4, Hepascore, FibroTest, Fibrometer, and TE for the staging of fibrosis in CHC, as compared to LB. The performance of these scores was similar, with slightly insignificant differences, as reported in other studies [24]. The AUROC of each studied test was comparable to those reported in the original publications [6][7][8][9][10][11][12]. For Fibrotest®, Hepascore®, Fibrometer®, and TE, the diagnostic performance was almost similar to those reported in meta-analyses [25][26][27]. Fibrometer performed better than simple tests in discriminating significant fibrosis and severe fibrosis. We tried to increase the diagnostic accuracy using combinations of simple serological markers with complex biomarkers or with TE. The associations were made on the basis of cost and accessibility. The possible combinations of markers were derived from multiple regression of the AUROCs.

Our results suggested that an algorithm combining Fibrometer, FibroTest, or TE with simple tests, such as APRI and or Fib-4, could improve the accuracy for significant and severe fibrosis and consequently decrease the need for biopsy. These combinations could be used reliably for the evaluation of fibrosis in HCV-infected patients, and this could avoid LB examination in most patients with CHC. The diagnostic performance of TE, FibroTest, and APRI based on AUROC values has been analyzed in many studies [15][25][28][29]. These studies did not show significant differences, although TE and FT usually tended to perform slightly better than APRI, but there are some studies that have suggested that FT provides a more accurate estimation of fibrosis in CHC than APRI [29]. Fibrotest was included in combinations of scores in order to increase the diagnostic accuracy, and the number of well-classified patients was 76% in combination with Fibrometer and Hepascore and 80% with APRI [30]. Another study reported a concordance of the combination of Fibotest, TE, and LB of 84% [31]. On the other hand, there are few studies that have included Fibrometer in a combined analysis of diagnostic performance.

New fibrosis indexes combining Fibrometer and Fibroscan have been developed for the diagnosis of clinically significant fibrosis (CSF-index) or severe fibrosis (SF-index). Their association provided a new fibrosis stage classification (CSF/SF classification): F0/1, F1/2, F2 ± 1, F2/3, F3 ± 1, and F4. This classification had high diagnostic accuracy (85.8% well-classified patients), significantly higher than the diagnostic accuracies of Fibrometer, Fibroscan, and Fibrotest [32]. Another study combined synchronous Fibrometer and TE, with a diagnostic accuracy of 91.9% for liver fibrosis staging, significantly higher than Fibrometer or TE alone, and with a higher percentage of avoided liver biopsies than previously published SAFE and Bordeaux algorithms [33]. Considering that Fibrometer performed well in our study group, we analyzed whether combinations that included this test could increase the diagnostic accuracy. The results revealed that adding Fibrometer to a simple inexpensive test would increase the performance of the biomarkers for discriminating significant and severe fibrosis. The combination of Fibrometer with APRI could be reliably used for the evaluation of fibrosis and consequently avoid LB, as demonstrated by the percentage by which the 2 tests agreed (90%) and were concordant with LB.

A similar observation is also valid for the combination of Fib-4 plus Fibrometer for the discrimination of significant fibrosis. With regard to severe and significant fibrosis, we found that not only was the combination of simple scores with Fibrometer useful for the diagnostic performance, the combination of APRI or Fib-4 with TE also increased the accuracy of the non-invasive methods. APRI and TE agreed on the discrimination of severe fibrosis in 74.5%, consistent with the results of liver biopsy in 85.7% of cases. In a similar analysis, which studied the performance of TE and FibroTest, Castera et al. obtained a concordance of 84% between these two methods and LB for significant fibrosis and 95% for severe fibrosis [31]. A very recently published study, comparing 9 of the well-known blood tests and TE, demonstrated a rate of 78% of well-classified patients with regard to significant fibrosis for the combination of APRI with Fibrometer. The association of Hepascore or FibroTest with APRI resulted in 80% well-classified patients; the percentage of theoretically avoided LB ranges from 58% to 62% for these tests [30]. When the study of non-invasive methods for assessment of liver biopsy becomes a concern, another important issue is the cost of different diagnostic methods. There are some studies that assessed the cost-effectiveness of non-invasive testing strategies in comparison with liver biopsy [34]. The authors reported cost savings of $770/person with the Fibrotest and $1120 with Fibroscan compared to liver biopsy, underlining the accesibility of non-invasive methods. However, the authors also reported that there is a decrease in diagnostic accuracy of 14% with Fibrotest and of 18% with Fibroscan compared to LB. Our study has some limitations. One is the length of the LB sample. In order to avoid misdiagnosis and the errors of interpretation, only biopsy specimens with more than 6 intact portal tracts were eligible for evaluation [35]. The diagnostic performance of TE also might have been underestimated. TE may provide a more accurate and reproducible picture of fibrosis stage than LB examination in patients with cirrhosis. Even if it has many advantages-it is painless, rapid, and easy to perform-unfortunately, it has some limitations, especially in the case of obesity and ascites. On the other hand, morphologically speaking, the increased grade of steatosis might be responsible for the false positive results of increased liver stiffness [12].

In summary, the analysis of the diagnostic performance of biomarkers for the assessment of liver fibrosis and transient elastography show that when isolated, these methods perform similarly with slightly differences, but a synchronous combination of a simple, inexpensive blood test that incorporates routine biochemical determinations with Fibrometer, FibroTest, or TE effectively increases the diagnostic performance of NIT. Improving the diagnosis accuracy, this type of combination could decrease the need for biopsy, but further studies are needed to assess the optimal combinations, based on cost-benefit. Although the combination of three tests had significant accuracy, this small increase does not justify the cost of the investigations.
